# Macular Thickness Measurements with Frequency Domain-OCT for Quantification of Retinal Neural Loss and its Correlation with Cognitive Impairment in Alzheimerʼs Disease

**DOI:** 10.1371/journal.pone.0153830

**Published:** 2016-04-22

**Authors:** Leonardo Provetti Cunha, Luciana Cheker Lopes, Luciana Virgínia Ferreira Costa-Cunha, Carolina Ferreira Costa, Leopoldo Antônio Pires, Ana Laura Maciel Almeida, Mário Luiz Ribeiro Monteiro

**Affiliations:** 1 Department of Ophtalmology, School of Medicine, Federal University of Juiz de Fora, Juiz de Fora, Minas Gerais, Brazil; 2 Juiz de Fora Eye Hospital, Minas Gerais, Juiz de Fora, Minas Gerais, Brazil; 3 Department of Neurology, School of Medicine, Federal University of Juiz de Fora, Juiz de Fora, Minas Gerais, Brazil; 4 Division of Ophtalmology, University of São Paulo Medical School, São Paulo, São Paulo, Brazil; International University of Health and Welfare, JAPAN

## Abstract

**Purpose:**

To evaluate the ability of frequency domain optical coherence tomography (fd-OCT) to estimate retinal neural loss in eyes with Alzheimer’s disease (AD). We also verified the existence of a correlation between AD-related cognitive impairment and macular and peripapillary retinal nerve fiber layer (RNFL) thickness measurements.

**Methods:**

fd-OCT scans were obtained from 45 eyes of 24 patients with AD and 48 control eyes. Peripapillary RNFL, macular full-thickness and segmented inner macular thickness parameters were calculated. The inner macular parameters included macular retinal nerve fiber layer (mRNFL) thickness, ganglion cell layer (GCL) plus inner plexiform layer thickness (GCL+), and RNFL plus GCL+ thickness (GCL++). The Mini-Mental State Examination (MMSE) was used to assess cognition in all subjects. The two groups were compared and the relationship between MMSE scores and fd-OCT measurements was verified.

**Results:**

Average, superior and inferior quadrant RNFL thickness parameters and all but one of the nine full-thickness macular measurements were significantly reduced in AD patients compared to controls. The segmented layers, GCL+ and GCL++ were significantly reduced in AD eyes. A significant correlation was found between most fd-OCT parameters (especially macular thickness measurements) and MMSE scores.

**Conclusions:**

Most fd-OCT peripapillary RNFL and macular full-thickness and segmented inner retinal layers parameters were reduced in AD eyes compared to controls. Moreover, neuronal loss, especially as reflected in macular parameters, correlated well with cognitive impairment in AD. Our results suggest that fd-OCT could be a potentially useful diagnostic tool in the evaluation and follow-up of AD patients.

## Introduction

Alzheimer’s disease (AD), the most common cause of degenerative dementia, is characterized by progressive cognitive deficits, including memory disturbances, aphasia, apraxia, agnosia and visual abnormalities [1,2]. Visual complaints in AD patients, including impairment of spatial contrast sensitivity, motion perception, color discrimination and visual loss, are usually attributed to lesions affecting specific areas of the brain, mainly the primary visual cortex [2–5]. However, there is evidence that anterior visual pathway impairment, involving predominantly retinal ganglion cells (RGC) and their fibers [6–8], also plays a role in visual dysfunction in AD [9,10].

Optical coherence tomography (OCT) is a non-invasive technology, which acquires cross-sectional images of retinal structures for the assessment of neural fundus integrity. Over the last years, OCT has become the most widely used technology to detect and quantify structural axonal damage in a range of optic nerve diseases. Axonal loss is usually quantified by OCT based on peripapillary retinal nerve fiber layer (RNFL) thickness measurements, allowing an indirect estimation of RGC layer impairment. In fact, many authors have shown RNFL thickness to be reduced in AD patients, with a significant correlation between RNFL thickness and deficits in visual function and cognition [8,11–18].

Axonal loss may also be detected through analysis of macular thickness measurements. Since RGCs and their fibers account for 30–35% of the total retinal thickness in the macular area, macular thickness measurements may be used to quantify RGC loss, as demonstrated for eyes with glaucoma, papilledema, compressive or demyelinating optic neuropathies [19–24] and AD [11,16,25–28]. More recently, improvements in OCT technology, especially the advent of frequency domain OCT (fd-OCT), have allowed substantial enhancements in ultrastructural evaluations of the macula, enabling the assessment not only of full-thickness retinal measurements but also of different retinal layers. Previous studies have shown that after segmentation of macular thickness measurements, a reduction in the RGC layer may be an important indicator of neural loss in different diseases including glaucoma, papilledema, chiasmal compression, multiple sclerosis and neuromyelitis optica [23,29–31] but few previous studies analyzed macular thickness measurements using fd-OCT in AD [16–18,32,33] patients and only one was performed to investigate the relationship between the severity of AD and full thickness or segmented inner retinal layer macular thickness parameters in patients with mild cognitive impairment [18].

The purpose of this study was therefore to evaluate the ability of fd-OCT to estimate neural loss in eyes with AD. We also verified the existence of a correlation between cognitive impairment in AD and macular and peripapillary RNFL thickness parameters.

## Methods

### Study design and subjects

In this cross-sectional, prospective study, participants were recruited from the Department of Neurology of the School of Medicine of the Federal University of Juiz de Fora (Minas Gerais, Brazil). The study protocol followed the principles of the Declaration of Helsinki and was approved by the Institutional Review Board. All participants (or their first-degree relatives) gave their informed written consent.

A total of 45 eyes from 24 patients (16 women) with AD and 48 eyes from 24 healthy controls (15 women) were evaluated. AD patients were diagnosed according to the guidelines of the National Institute of Neurological and Communicative Disorders and Stroke and the Alzheimer’s Disease Association (“NINCDS-ADRDA”) [1].

Each patient underwent a full neurological examination and magnetic resonance imaging (MRI) of the brain to rule out alternative diagnoses. The Mini-Mental State Examination (MMSE) [34] was used to assess cognition in AD and control subjects.

The control group was made of healthy hospital staff volunteers or patients admitted for routine eye examination, mainly for refractive errors. Patients and controls underwent a detailed ocular examination, including measurement of visual acuity (VA), slit-lamp examination of the anterior and posterior segments of the eye, intraocular pressure (IOP) measurement by Goldmann applanation tonometry, central corneal thickness measurement and fundus examination.

Study group criteria for inclusion were: best-corrected VA of at 20/40 or better in at least one eye (patients) and 20/20 in both eyes (control group), spherical refraction within ± 5 diopters, cylinder correction within ±4D, and IOP below 22 mm Hg. The exclusion criteria for both patients with AD and controls were history of systemic arterial hypertension, diabetes mellitus, clinical findings of glaucomatous or other optic neuropathies, optic disc anomaly, history of IOP elevation, age-related macular degeneration or other maculopathies (e.g. epiretinal membrane, macular hole), media opacity (except for mild nuclear sclerosis). For patients with AD severe dementia preventing adequate examination was also an exclusion criterion.

### Optical coherence tomography

All subjects underwent fd-OCT scanning using commercially available equipment (3D OCT-2000, software version 8.11, Topcon Corp., Tokyo, Japan) and an ophthalmic evaluation on the same day. Images were reviewed by the authors regarding quality and only those with good quality were included. A set of three high-quality optic nerve head (ONH) and macular images was obtained in a raster pattern covering a 6x6 mm area with a scan density of 512 × 128 pixels (27 000 A scans/s). Inner macular layer thickness was based on a set of three high-definition fd-OCT images centered on the fovea in a raster pattern covering a 7x7 mm area with a scan density of 512 (vertical) × 128 (horizontal) pixels. The equipment measured a 6x6 mm area centered on the fovea using built-in software.

Criteria for acceptable 3D OCT-2000 fundus images included (i) no large eye movements, defined as an abrupt shift completely disconnecting a large retinal vessel, (ii) consistent signal intensity level across the scan, and (iii) no black bands (caused by blinking) throughout the examination, as previously described in this study [21].

Peripapillary RNFL, macular full-thickness and inner macular thickness parameters were automatically calculated by the equipment’s software. A circular (*Ø* = 3.4 mm) map drawn around the ONH was used to measure peripapillary RNFL average thickness (360°), temporal quadrant thickness (316° to 45°), superior quadrant thickness (46° to 135°), nasal quadrant thickness (136° to 225°) and inferior quadrant thickness (226° to 315°).

**Fig 1:**
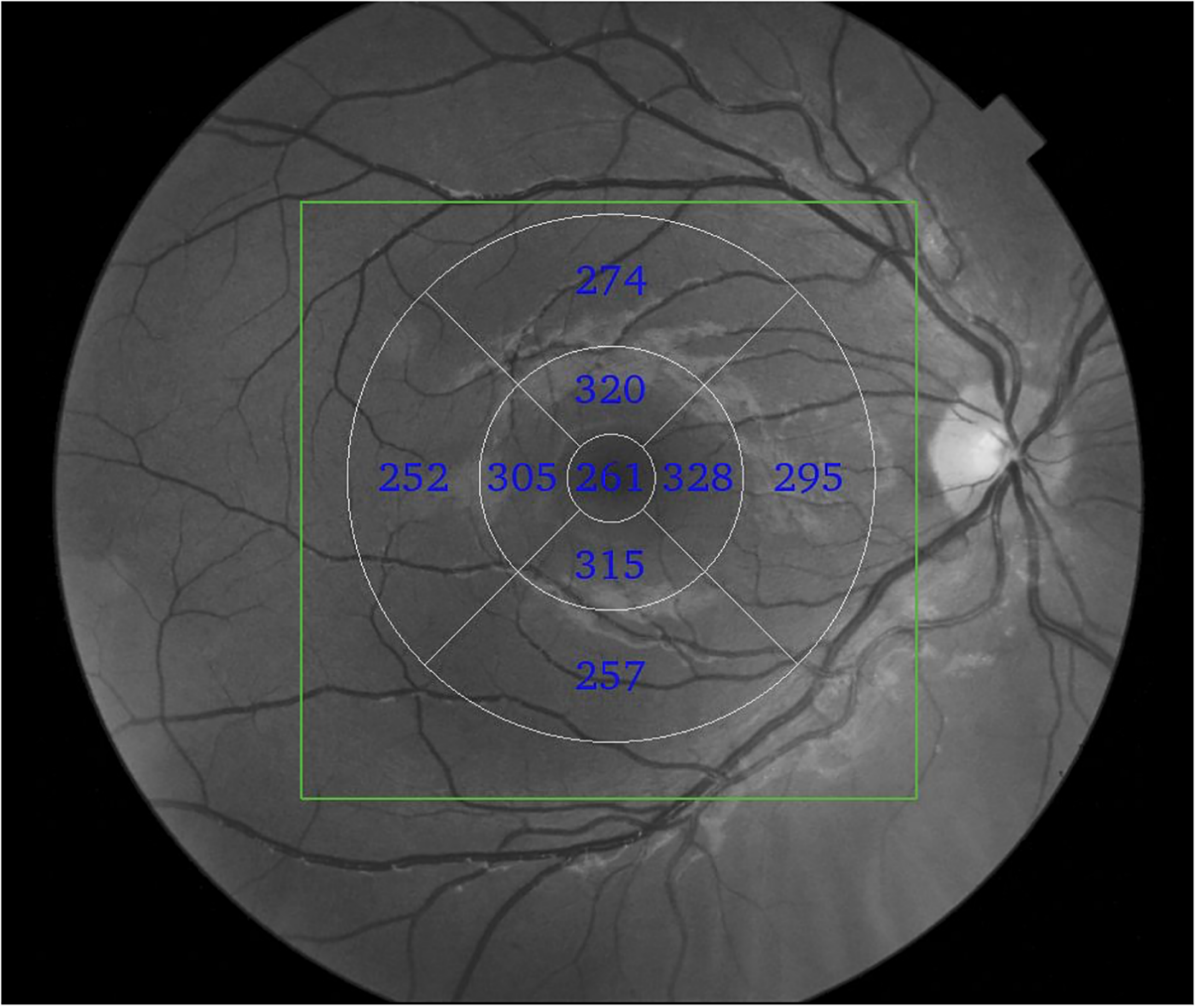
Example of fd-OCT generated macular full-thickness measurements of the right eye. The green rectangle demarcates the scanned area (6x6mm). The nine macular full-thickness sectors were based on Early Treatment Diabetic Retinopathy Study map.

The macular full-thickness measurements were based on the Early Treatment Diabetic Retinopathy Study map. The parameters registered in this study were superior outer macular thickness, inferior outer macular thickness, temporal outer macular thickness, nasal outer macular thickness, superior inner macular thickness, inferior inner macular thickness, temporal inner macular thickness, nasal inner macular thickness, average macular thickness and fovea (Fig 1). Average macular thickness corresponded to the weighted average of the sectoral macular thickness measurements excluding the fovea, as previously described in this study [21].

**Fig 2:**
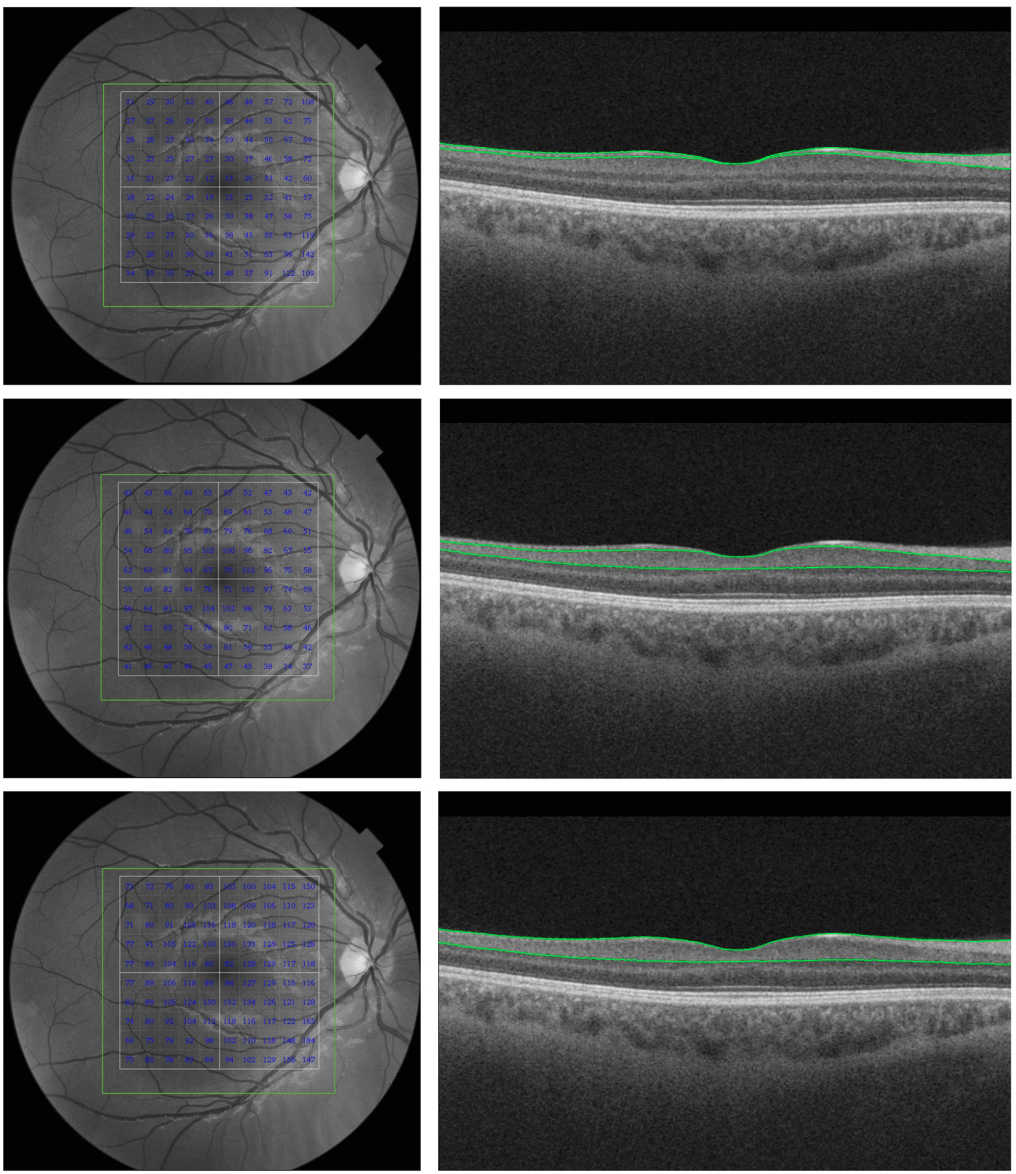
Example of fd-OCT-generated inner macular thickness measurements. Left: scanned area (7x7 mm). The inner retinal layers thickness measurements are show in each 10x10 grid cells. The total analyzed area corresponded to 6x6 mm. Right: horizontal fd-OCT B scan through the fovea. The green lines correspond to the boundaries of the inner retinal layers identified during the segmentation process. The first row represents macular RNFL (mRNFL) thickness measured from the internal limiting membrane (ILM) to the inner boundary of the ganglion cell layer (GCL). The second row represents GCL + inner plexiform layer (IPL) thickness measured from the inner boundary of the GCL to the outer boundary of the IPL (GCL+). The third row represents mRNFL + GCL + IPL measured from the ILM to the outer boundary of the IPL (GCL++).

The inner macular layer thickness measurements were automatically registered in a square pattern (6x6 mm) from within the 7 X 7 mm scanned area. In each B-scan, the boundaries between the anatomical inner retinal layers of the macular area were automatically defined by built-in software (Fig 2). An experienced examiner evaluated the boundaries automatically defined by the software in each scan and repeated the entire acquisition when boundary errors were present. Manual corrections of the segmentation were not performed. The parameters registered in this study were: 1) average macular retinal nerve fiber layer (mRNFL) thickness, 2) average ganglion cell layer (GCL) plus inner plexiform layer (IPL) thickness (referred to as GCL+), 3) and average RNFL plus GCL+ (GCL plus IPL) thickness (referred to as GCL++) (Fig 2).

### Data analysis and statistics

Descriptive statistics included mean ± standard deviation (SD) for normally distributed variables, and median and first and third quartiles for non-parametric variables.

Data from the two groups was compared generalized estimating equation (GEE) models to compensate for the fact that in some patients and controls both eyes were included in the study. GEE models were therefore used to adjust for within-patient inter-eye correlations. We used receiver operating characteristic (ROC) curves to describe the discrimination ability of fd-OCT parameters. Areas under the ROC curves (AUCs) were compared using DeLong et al.’s method [35]. Association between fd-OCT and MMSE was assessed using Pearson’s correlation coefficients. Results were considered significant when *p*<0.05 was obtained.

## Results

Table 1 shows the demographic characteristics and MMSE scores of patients and healthy controls. A total of 93 eyes were evaluated, of which 45 were from 24 patients with AD and 48 were from 24 healthy age-matched controls. The mean age ± SD was 74.80 ± 6.25 years (range: 62–84) in AD patients and 72.25 ± 7.31 (range: 61–83) in the control group (*p* = 0.26, Student’s *t*-test). The mean MMSE score ± SD was 17.02 ± 5.20 (range: 2–24) in AD patients and 29.08 ± 1.62 (range: 25–30) in the control group. The MMSE score was significantly lower in AD patients (*p*<0.001, Student’s *t*-test). The fundus examination was unremarkable in all eyes. A total of 3 eyes of 3 patients were excluded, two due to media opacity (severe senile cataract) and one because of OCT-detected vitreous macular traction on the fovea.

**Table 1:** Demographic characteristics of AD patients and healthy controls.

	*Alzheimer’s disease*	*Controls*	*p*
**Subjects**	24	24	
**Eyes studied**	45	48	
**Sex M/F**	08/16	09/15	
**Age, y, mean (SD)**	74.80 (6.25)	72.25 (7.31)	0.26*
**SE power, mean (SD)**	0.18 (1.22)	0.50 (1.32)	0.23
**MMSE score**	17.02 (5.20)	29.08 (1.62)	*0*.001*
**Course of AD, y, mean (SD)**	2.68 (2.83)		
**IOP mmHg (SD)**	14.02 (0.52)	14.77 (0.60)	0.349**

M = male; F = female; y = years; SD = standard deviation; SE = spherical equivalent; MMSE = Mini-Mental State Examination, AD = Alzheimer’s disease; IOP = intra ocular pressure; Significant values in italics.

* Student’s *t* test;

** GEE.

Table 2 shows fd-OCT parameters of AD patients and controls. Average thickness, superior and inferior quadrant RNFL thickness parameters were significantly smaller in AD eyes than in controls. All macular full-thickness parameters, with the exception of inferior outer macular segment measurements, were significantly smaller in AD eyes. ROC curve areas and sensitivities at fixed specificities are shown in Table 2. The macular thickness of the superior (AUC = 0.82), temporal (AUC = 0.83), inferior (AUC = 0.86) and nasal (AUC = 0.83) inner segments had the largest AUCs. GCL+ and GCL++ macular thickness parameters were significantly smaller in eyes of AD patients (*p*<0.05), with AUCs of 0.71 and 0.72, respectively. mRNFL thickness was smaller in AD eyes than in control eyes, but the difference did not reach statistical significance (p = 0.06).

**Table 2:** Mean values (SD) of frequency domain optical coherence tomography parameters of peripapillary RNFL thickness and full and inner macular thickness (in μm), with areas under the receiver operating characteristic curves (AUC) and sensitivities at fixed specificities.

*Parameter*	*Alzheimer’s disease (n = 45)*	*Controls(n = 48)*	p*	*AUC (SE)*
***Peripapillary RNFL***				
**Average thickness**	93.75 (13.42)	102.96 (9.19)	*<0*.*001*	0.70 (0.05)
**Superior**	110.88 (19.76)	122.12 (15.55)	*0*.*01*	0.66 (0.06)
**Temporal**	66.91 (15.66)	72.69 (9.16)	0.07	0.59 (0.06)
**Inferior**	112.38 (23.25)	130.10 (12.78)	*<0*.*001*	0.77 (0.05)
**Nasal**	82.64 (17.82)	86.46 (13.61)	0.34	0.56 (0.06)
***Macula (full thickness)***				
**Average thickness**	257.89 (17.11)	273.45 (13.14)	*<0*.*001*	0.74 (0.05)
**Superior inner**	279.86 (21.10)	303.75 (13.45)	*<0*.*001*	0.82 (0.05)
**Temporal inner**	269.84 (18.31)	293.45 (15.86)	*<0*.*001*	0.83 (0.04)
**Inferior inner**	270.17 (20.61)	300.10 (17.03)	*<0*.*001*	0.86 (0.04)
**Nasal inner**	281.75 (20.65)	308.12 (16.25)	*<0*.*001*	0.83 (0.04)
**Superior outer**	251.80 (20.35)	264.08 (14.78)	*0*.*01*	0.68 (0.06)
**Temporal outer**	242.15 (17.33)	255.52 (14.79)	*0*.*001*	0.72 (0.05)
**Inferior outer**	252.26 (19.60)	260.62 (17.16)	0.089	0.62 (0.06)
**Nasal outer**	246.60 (21.51)	280.50 (15.55)	*0*.*02*	0.70 (0.05)
**Fovea**	221.38 (27.86)	246.62 (25.26)	*0*.*001*	0.75 (0.05)
***Macula (inner layer)***				
**mRNFL**	32.51 (5.96)	34.77 (3.12)	0.06	0.60 (0.06)
**GCL+**	63.24 (7.60)	69.00 (6.09)	*0*.*003*	0.71 (0.05)
**GCL++**	95.66 (11.69)	103.85 (7.73)	*0*.*002*	0.72 (0.05)

* = Generalized estimated equations. Significant values are in italics.

**Fig 3:**
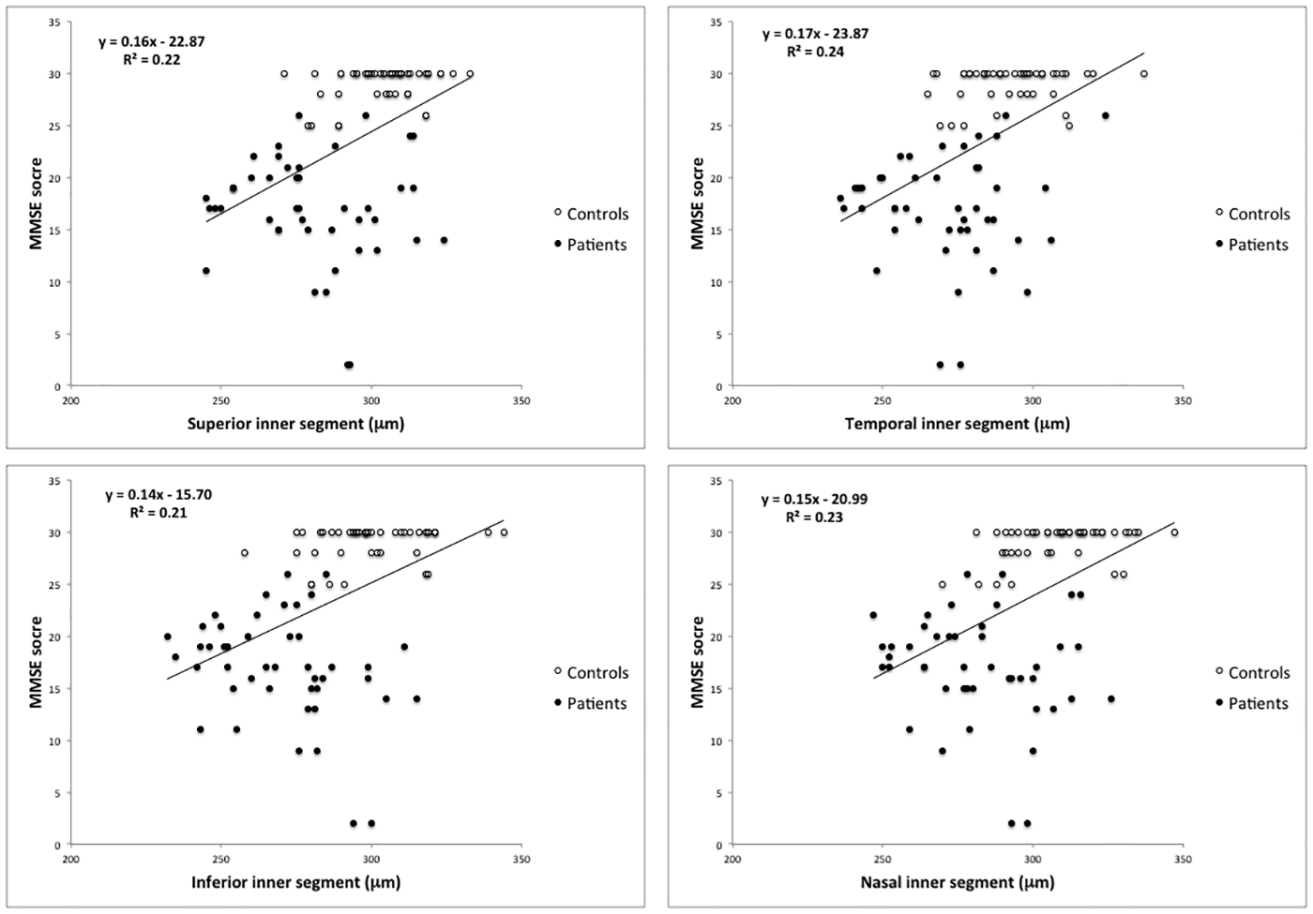
Scatter plots of MMSE scores plotted against fd-OCT measurements of the 4 inner macular segments. The parameters with the strongest correlation were selected.

Table 3 shows the correlation between MMSE scores and average/sectorial macular and peripapillary RNFL thickness parameters. Significant correlations between fd-OCT and MMSE scores were found for most macular and peripapillary RNFL thickness measurements. The three most significant correlations between peripapillary RNFL thickness and MMSE scores were with average thickness (r = 0.33), inferior quadrant (r = 0.37) and superior quadrant (r = 0.22) thickness measurements. The three most significant correlations between macular full-thickness measurements and MMSE scores were temporal inner (r = 0.49), nasal inner (r = 0.48) and fovea (r = 0.46). All three inner macular thickness parameters (mRNFL, GCL+ and GCL++) were significantly correlated with MMSE scores (r = 0.30, 0.33 and 0.37, respectively). Fig 3 shows the results of the linear regression analysis of the best performing fd-OCT macular thickness parameters (4 inner macular segments) and MMSE scores.

**Table 3:** Relationship between fd-OCT parameters and mini-mental state examination (MMSE) scores.

*Parameter*	MMSE score	*p*
***Peripapillary RNFL***		
**Average thickness**	0.33	*0*.*001*
**Superior**	0.24	*0*.*019*
**Temporal**	0.14	0.174
**Inferior**	0.37	*<0*.*001*
**Nasal**	0.15	0.*132*
***Macula (full thickness)***		
**Average thickness**	0.34	*0*.*001*
**Superior inner**	0.47	*<0*.*001*
**Temporal inner**	0.49	*<0*.*001*
**Inferior inner**	0.46	*<0*.*001*
**Nasal inner**	0.48	*<0*.*001*
**Superior outer**	0.19	0.187
**Temporal outer**	0.30	*0*.*003*
**Inferior outer**	0.14	0.141
**Nasal outer**	0.31	*0*.*002*
**Fovea**	0.47	*<0*.*001*
***Macula (inner layer)***		
**mRNFL**	0.30	*0*.*003*
**GCL +**	0.33	*0*.*001*
**GCL ++**	0.37	*<0*.*001*

Pearson’s correlation coefficients. Significant values in italics.

## Discussion

The results of the present study show that most peripapillary RNFL and macular thickness parameters were significantly reduced in eyes with AD. Inner retinal layer measurements were also significantly reduced, a previously unreported finding for this patient population. Moreover, most fd-OCT parameters correlated significantly with MMSE scores.

Our findings match the results of several other studies reporting peripapillary RNFL loss in AD patients [8,11,13,15,36]. More specifically, the observed reduction in average, superior quadrant and inferior quadrant peripapillary RNFL thickness is supported by studies identifying the superior and inferior quadrants as the most frequently affected in AD [12,25,36,37]. This is consistent with the histopathological findings of Hinton et al. [9] evidencing RGC loss and optic nerve impairment in eyes of AD patients. Subsequent analyses confirmed these findings and showed the loss to involve predominantly large RGCs (M-cells), contributing to axonal loss in patients with AD [10]. Taken together, histopathological and OCT findings indicate that, in addition to visual cortex degeneration, the anterior visual pathway is also affected in AD patients.

We also demonstrated that macular parameters are markedly reduced in eyes with AD. Only two previous studies have evaluated macular parameters in AD patients [11,25]. Iseri et al. [11] evaluated 14 patients with AD using td-OCT and found that full-thickness macular measurements were reduced in 6 of the 9 thickness parameters (*p*<0.05). Moschos et al. [25] also evaluated the central macular thickness in 30 AD patients using td-OCT and found it to be significantly reduced in AD compared to controls. Other macular thickness measurements were not reported. Our study, using high-resolution fd-OCT, confirms these findings in that all but one (inferior outer segment) macular thickness measurements were significantly reduced in AD eyes compared to controls. It is noteworthy that the inner segments, including the fovea, were the most affected parameters in our study (Table 2), suggesting a pattern of neuronal loss in patients with AD. These OCT findings are supported by a histological analysis by Blank et al. showing a 25% overall decrease in the number of neurons in the GCL at the level of the fovea and parafoveal area of the retina in AD patients [38]. Other authors have provided evidence of AD-related RGC loss in AD patients. Thus, Alexandrov et al. [39] described amyloid-mediated inflammatory degeneration with accumulation of amyloid peptides in the retina and brain of AD patients, showing that amyloid accumulation may contribute to retinal damage in these patients. Likewise, Dutescu et al. [40] reported amyloid accumulation in the GCL and inner nuclear layer (INL) of transgenic mice.

The recent introduction of fd-OCT (which produces images of higher resolution than time-domain OCT) has allowed the segmentation of different layers of the retina in the macular area, including the mRNFL, the GCL and the INL. This approach can be very useful in the evaluation of AD patients, to better understand which retinal structures are affected and possibly to confirm *in vivo* RGC degeneration observed in postmortem studies [39] and suggested in electrophysiological tests [8]. Currently, AD patients are monitored through clinical examinations and cognitive tests. MRI of the brain is the only objective tool available to assess these patients. Therefore, fd-OCT-detected changes in the inner retinal layers suggestive of neuronal impairment are of great clinical relevance to the diagnosis and monitoring of AD patients. Our data is important in this regard since it clearly showed significantly reduced GCL thickness measurements confirming that RGC layer plays an important role on reduced measurements of macular full-thickness parameters. However, while our data using segmented retinal layers did document mRNFL and GCL reduction in such patients, the difference in significance and the ROC curve values (ranging from 0.60 to 0.72) were not superior to other full-thickness macular parameters, particularly the fovea, and the inner full-thickness circular measurements (ROC curves ranging from 0.62 and 0.86). Since the current software provides only average macular RNFL, GCL+ and GCL++ measurements it is possible that not analyzing more specifically the central areas of the macula (using the whole 6x6 mm average data instead) prevented us from detecting more localized disease involvement in the central area including the fovea. Furthermore it is important to point out that the current software did not allow segmenting outer retinal layers that may also be affected in AD. Using multifocal electroretinography, Moschos et al. [25] observed reduced P1 amplitudes in the foveal and parafoveal area, suggesting a functional disorder of the outer retina in the central macula. Further studies are therefore needed to understand possible involvement of other retinal layers and thee topography of such involvement evaluating segmented retinal layers in more localized areas in the fovea.

Another purpose of our study was to verify the existence of a correlation between structural retinal degeneration (fd-OCT parameters) and the cognitive impairment (MMSE scores) in AD. As shown in Table 3, our findings confirmed a significant correlation between MMSE scores and several fd-OCT parameters. Interestingly, the most significant correlations were those of the four (superior, inferior, nasal and temporal) inner macular segments, the fovea and GCL++ (*p*<0.001), reflecting the most affected parameters in our patients. Using time-domain OCT, Iseri et al. [11] also found a significant correlation between total macular volume and MMSE score in AD patients (r = 0.696).

Our study has some limitations. First, our sample was relatively small due to the exclusion of patients with other ocular or systemic diseases. The incidence of systemic hypertension, glaucoma and macular degeneration is known to be higher in elderly patients. Second, due to the inclusion of patients at all stages of AD, visual function was not evaluated. Subjective methods, such as VA and visual field examination, are not reliable in advanced stages of AD. Finally, as pointed out above, the study was limited by the inability of fd-OCT to assess all retinal layers, especially those of the outer retina.

In summary, in this study we not only confirmed that macular thickness measurements are markedly reduced in AD patients but, for the first time, quantified the loss of neural cells in the inner retinal layers of the macular area. The loss reflects AD-related neuronal degeneration of the retina in a characteristic pattern predominantly affecting the central macular area. Moreover, neuronal loss (especially as expressed in macular parameters) correlated well with cognitive impairment in AD. Thus, our results suggest that fd-OCT could be used as a swift and non-invasive diagnostic tool in the routine evaluation and follow-up of AD patients, allowing a more comprehensive approach to this disease.
